# Safe Tummy Tuck: Anatomy and Strategy to Avoid Injury to the Lateral Femoral Cutaneous Nerve During Abdominoplasty

**Published:** 2015-06-17

**Authors:** S. Chowdhry, J. Davis, T. Boyd, J. Choo, R. M. Brooks, S. S. Kelishadi, J. P. Tutela, D. Yonick, B. J. Wilhelmi

**Affiliations:** Division of Plastic Surgery, University of Louisville, Louisville, Ky

**Keywords:** abdominoplasty, safety, lateral femoral cutaneous nerve, plastic surgery, tummy tuck

## Abstract

**Background:** Abdominoplasty is one of the most common aesthetic procedures performed in the United States. While poor contour and unsatisfactory cosmetic result have been recognized, neuropathic pain from lateral femoral cutaneous nerve injury has been poorly described. We aim to improve outcomes by using an anatomical study to develop a strategy to avoid injury to the lateral femoral cutaneous nerve in abdominoplasty. **Methods:** Twenty-three fresh cadaver abdomens were dissected to evaluate the course of the lateral femoral cutaneous nerve, using 2.5× loupe magnification. Measurements were taken from the nerve to the anterior superior iliac spine and from the pubic symphysis to the lateral femoral cutaneous nerve. Recordings of the relationship of the nerve to the inguinal ligament and depth at scarpa's fascia were also made. Statistical analysis was performed to find average distances with a standard deviation. **Results:** On average, the distance from the lateral femoral cutaneous nerve to the anterior superior iliac spine was 3.62 (SD = 1.32) cm and 13.58 (SD = 2.41) cm from the pubic symphysis in line with the inguinal ligament. The lateral femoral cutaneous nerve was found at the inguinal ligament 80% of the time and 20% of the time superior to the ligament and always deep to scarpa's fascia. **Conclusion:** Abdominoplasty carries a high patient and surgeon satisfaction rate. The plastic surgeon is continuously challenged to identify ways to improve outcomes, efficiency, and morbidity. Minimal and careful dissection in the area around 4 cm of the anterior superior iliac spine in addition to preserving scarpa's fascia near the inguinal ligament may serve as key strategies to avoiding lateral femoral cutaneous nerve injury.

Abdominoplasty is one of the most common aesthetic procedures performed in the United States today. Close to 150,000 procedures were performed in 2010, which is almost 3 times the volume performed in 1997.^1^ As the fourth most commonly performed cosmetic procedure, the plastic surgeon needs to be familiar with not only methods to produce an aesthetically pleasing result but also its potential complications. While poor contour, deformity, and unsatisfactory cosmetic result have been recognized, neuropathic pain from lateral femoral cutaneous nerve (LFCN) injury has been poorly described. The importance of this injury has been demonstrated to be significant by patients due to the constant neuropathic pain.

As this complication has the potential for a significantly compromised outcome, the authors outline a study to improve surgical safety. Through identifying a potential complication of a common procedure, this study aims to improve patient quality of life by using an anatomical study to develop a strategy to avoid injury to the LFCN in abdominoplasty surgery.

## METHODS

**Figure 1:**
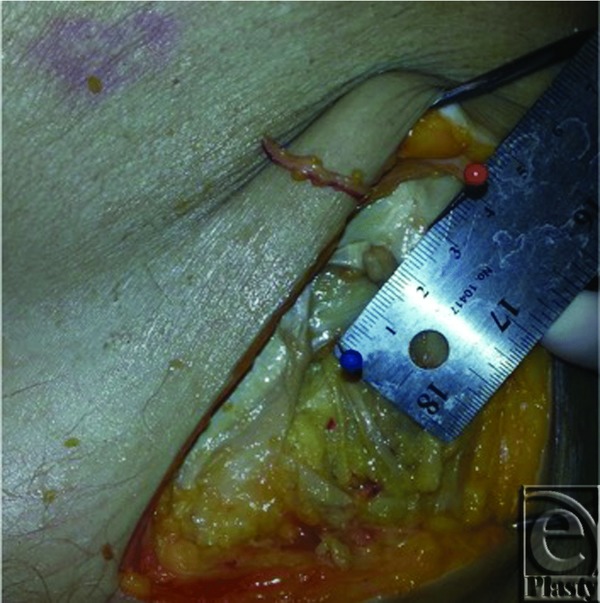
Dissection of the left LFCN showing distance from ASIS (red pin) to LFCN (blue pin). LFCN indicates lateral femoral cutaneous nerve; ASIS, anterior superior iliac spine.

**Figure 2:**
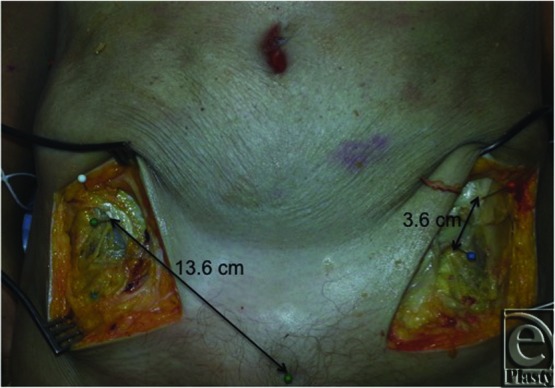
Dissection of bilateral LFCNs showing distance from the pubic symphysis to LFCN and ASIS to LFCN. LFCN indicates lateral femoral cutaneous nerve; ASIS, anterior superior iliac spine.

At the University of Louisville Fresh Tissue Lab, 25 cadaver hemiabdomens were dissected to evaluate the course of the LFCN, using 2.5× loupe magnification. Measurements were taken from the nerve to the anterior superior iliac spine (ASIS) and from the pubic symphysis (PS) to the LFCN. Recordings of the relationship of the nerve to the inguinal ligament and depth at scarpa's fascia were also made. These data were recorded, and statistical analysis was performed to find average distances with a standard deviation (Figs 1 and 2).

## RESULTS

On average, the distance from the LFCN to the ASIS was 3.62 (SD = 1.32) cm and 13.58 (SD = 2.41) cm from the PS in line with the inguinal ligament (Table 1). The LFCN was found at the inguinal ligament 80% of the time and 20% of the time superior to the ligament and always deep to scarpa's fascia.

## DISCUSSION

Aesthetic abdominoplasty procedures aim to flatten the abdomen through excision of excess skin and fat while contouring the torso through fascial plication. Concomitant liposuction has been shown to be a safe and effective adjunct to traditional abdominoplasty.^2^^-^5

Complications of abdominoplasty range from embolic events to wound complications to poor aesthetic outcomes.^5^^-^13 Numerous studies have identified techniques to reduce the most common complication secondary to abdominoplasty: seroma formation. While tissue glues and perforator ligation may play a role in reduced seroma formation, progressive tension or quilting sutures and suction drainage seem to have an advantage in seroma reduction.^14^^-^17 General medical comorbidities such as diabetes mellitus and smoking appear to play an increased role in wound-healing complications.^9^

Frequently, patients will describe a temporary numbness of their abdominal wall after abdominoplasty. However, as the LFCN innervates the anterior and lateral thigh, injury to this nerve can result in numbness and pain in this dermatomal distribution.

Lateral femoral nerve injury is a known complication of abdominoplasty, as several studies have identified its occurrence in the literature. Although its incidence of occurrence is about 1.36%, this is the most common nerve injury identified with cosmetic abdominoplasty.^1^^,^^4^^,^^10^^,^^12^^,^^14^^,^^18^^,^^19^ Review of the literature suggests that only 25% of patients who sustain an injury to the LFCN will recover.^14^^,^^18^

As abdominoplasty procedures serve to improve patient quality of life, complications that interfere with that goal are poorly tolerated. While tissue glues, perforator ligation, chemoprophylaxis, drains, and anesthetic adjuncts can aid in reducing many complications of cosmetic abdominal surgery, there is no substitute for anatomical mastery to meet the challenge of minimizing the poorly tolerated complication of LFCN injury.

**Figure 3:**
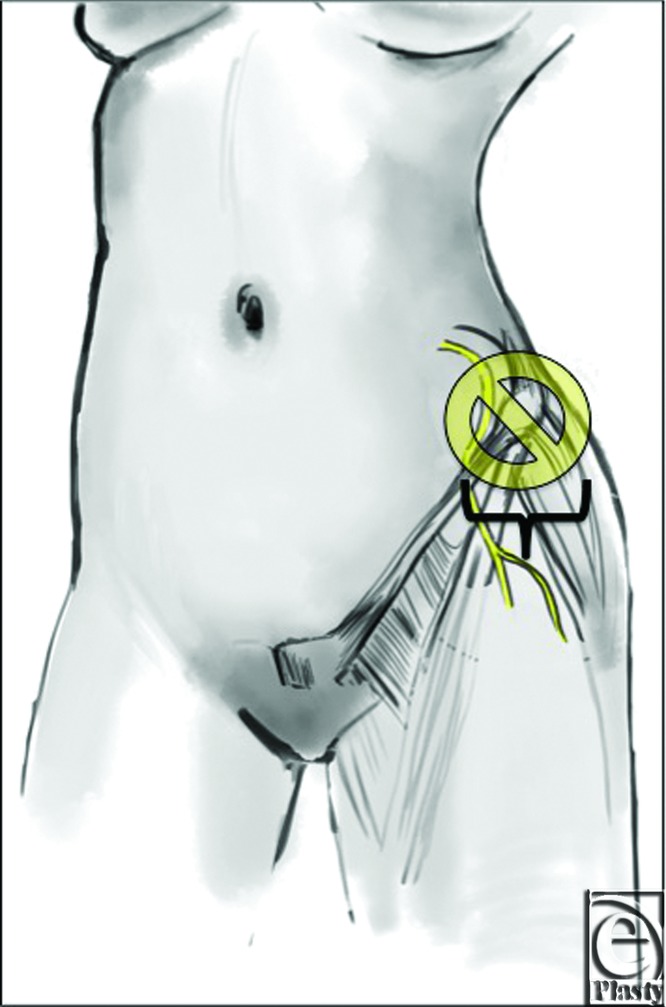
Diagram of the unsafe zone of dissection around the anterior superior iliac spine and the inguinal ligament.

Topographic landmarks of the ASIS, PS, xiphoid, and fascial system knowledge can aid the surgeon in safely performing common abdominal cosmetic procedures. Minimal and careful dissection in the area around 4 cm of the ASIS in addition to preserving scarpa's fascia near the inguinal ligament may serve as key strategies to avoiding LFCN injury (Fig 3).

Anatomy of the abdominal wall proves to be predictable and elegant. Comprehensive knowledge of neurovascular anatomy, lymphatics, and fascial planes may prove critical to excellent results. Although the incidence of this injury is relatively small, the potential for a significant complication should be outlined by the plastic surgeon in an appropriate preoperative discussion.

## CONCLUSION

As one of the most commonly performed procedures, abdominoplasty generally carries a high patient and surgeon satisfaction rate. The plastic surgeon is continuously challenged to identify ways to improve outcomes, efficiency, and morbidity. Knowledge of the location of the LFCN may aid the surgeon in attaining these goals.

## Figures and Tables

**Table 1:** Distances from ASIS to LFCN at the level of the inguinal ligament and the distance from PS to LFCN^a^

Cadaver #	Gender	Side	LFCN-ASIS	LFCN-PS
1	M	L	3.5	12.5
2	M	R	4	12.9
3	M	L	2.4	12
4	M	L	2	12
5	M	R	2	11.2
6	M	L	3	13.6
7	M	R	3	15.1
8	M	L	2	17
9	F	R	2	16.5
10	M	R	4	13.1
11	F	L	3	13.5
12	F	R	5	13
13	M	R	6.8	19.7
14	M	L	3	14.5
15	M	R	2.3	16.5
16	F	L	4	11
17	F	R	4.5	12.5
18	F	L	4.5	12.5
19	M	R	6.5	8.5
20	F	R	5	15.5
21	M	L	3.5	14.5
22	M	R	4.5	16.5
23	M	R	3.5	12
24	F	L	2.5	11.8
25	F	R	4	11.7
Average			3.62	13.58
SD			1.32	2.41
*n*			25	25

Distances from the Anterior Superior Iliac Spine (ASIS) to the Lateral Femoral Cutaneous Nerve (LFCN) at the level of the Inguinal Ligament and the distance from the Pubic Symphasis (PS) to the LFCN.
